# Complete Genomic Sequences of Two Aeromonas hydrophila Isolates Derived from Diseased Fish in South Korea

**DOI:** 10.1128/mra.00786-22

**Published:** 2022-12-08

**Authors:** Diem Tho Ho, Yoonhang Lee, Nameun Kim, HeyongJin Roh, Ahran Kim, Do-Hyung Kim

**Affiliations:** a Department of Aquatic Life Medicine, College of Fisheries Science, Pukyong National University, Busan, South Korea; Montana State University

## Abstract

Aeromonas hydrophila is a Gram-negative pathogen that is associated with motile aeromonad septicemia in various fish species. Here, we report the complete genomic sequences of two A. hydrophila isolates derived from diseased fish in South Korea.

## ANNOUNCEMENT

Aeromonas hydrophila is an opportunistic pathogen in a variety of animals, including humans (1, 2) and is the causative agent of motile aeromonad septicemia in different fish species worldwide (3, 4). Here, we report the complete genomic sequences of strains AC133 and AC185, isolated by swabbing the internal section of a kidney obtained aseptically from hemorrhagic and/or exophthalmic crucian carp (Carassius carassius) and American eel (Anguilla rostrata), respectively, which were collected from fish farms in South Korea. The isolates were inoculated onto tryptic soy agar (TSA) (Oxoid, Thermo Fisher Scientific, USA) and incubated at 28°C for 24 h. Colonies identified as A. hydrophila by species-specific multiplex PCR targeting the *gyrB* and 16S rRNA genes as described previously (5) were stored in brain heart infusion broth (BHIB) (Merk, Darmstadt, Germany) supplemented with 15% glycerol at −80°C.

A single colony of each A. hydrophila strain grown on TSA was inoculated into BHIB and incubated at 28°C for 24 h. Genomic DNA was extracted using the Wizard DNA purification kit (Promega, USA) and quantified using a Qubit v3.0 fluorometer (Thermo Fisher Scientific) according to the manufacturer’s protocol. Whole-genome sequencing was performed using an RS II instrument (Pacific Biosciences [PacBio], USA). Library preparation was constructed using the PacBio DNA template prep kit 1.0 and BluePippin size-selection system according to the manufacturer’s instructions. The reads were filtered (minimum subread length, 500 bp; minimum polymerase read quality, 0.80) and *de novo* assembled using PacBio single-molecule real-time analysis 2.3.0 based on the HGAP2 protocol (Pacific Biosciences). Gene prediction was conducted using tRNAscan-SE (6) for tRNA search, Rfam (7) for rRNA and noncoding RNA search, and Prodigal (8) for coding sequence (CDS) search. The predicted genes were functionally annotated by homology search against the Kyoto Encyclopedia of Genes and Genomes (9), SEED (10), Swiss-Prot (11), and eggNOG (12) databases. Default settings were used for all software.

The sequencing and annotation results are summarized in Table 1. A circular chromosome was confirmed by Unicycler v0.4.9 (13) and rotated to *dnaA* as the start position. No plasmid was found in both isolates. The genome of AC133 and AC185 consisted of 5,037,528 and 4,963,702 bases with an overall G+C content of 60.9% and 61.3%, containing 4,475 and 4,400 CDSs, 125 and 126 tRNAs, and 31 and 31 rRNA genes, respectively. Strains AC133 and AC185 showed 96.8% and 97.04% average nucleotide identity analysis with type strain A. hydrophila ATCC 7966 (GenBank CP000462.1), respectively, using Edgar 3.0 (14). Various virulence-related genes, including those responsible for type IV pili, type II, and type VI secretion systems (15–17), were identified, based on the Virulence Factor Database (18). A BLAST search using the Comprehensive Antibiotic Resistance Database (19) (≥95% identity) revealed that both isolates harbor one antibiotic resistance-related gene (ARG), *bla*_OXA-726_, while additional multiple ARGs, namely, *bla*_TEM-1_, *bla*_CTX-M-3_, *cepS*, *catII*, *mphA*, *sul1*, *aadA2, aac(3)-IId, aac(6')-Ib10, aph(6)-Id, aph(3'')-Ib*, *aph(3')-Ia*, and *tet(D)*, were identified only in strain AC185. Both AC133 and AC185 exhibited no inhibition zones relative to ampicillin and amoxicillin, and AC185 was also resistant to cefoperazone, cefotetan, chloramphenicol, erythromycin, trimethoprim-sulfamethoxazole, gentamicin, and oxytetracycline using disk diffusion assays based on Clinical and Laboratory Standards Institute guidelines (20). These data were in a close agreement with the genotypic differences between the strains. The genome sequences presented here could improve understanding about the virulence and antibiotic resistance of A. hydrophila.

**TABLE 1:** Summary of genome data of the isolates

Strain	No. of reads	Coverage (×)^*a*^	*N*_50_ value (bp)	No. of contigs	Total length (bp)	G+C content (%)	No. of protein-coding genes	No. of rRNAs	No. of tRNAs	No. of plasmids
AC133	158,140	310	14,458	1	5,037,528	60.9	4,475	31	125	0
AC185	106,152	242	17,464	1	4,963,702	61.3	4,400	31	126	0

a Rounded to the nearest whole number.

### Data availability.

Raw sequences of A. hydrophila AC133 and AC185 were submitted to NCBI SRA with accession numbers SRR20950404 and SRR20950142, respectively; under BioProject number PRJNA812366; and under BioSample numbers SAMN26377313 and SAMN26377314, respectively. The complete sequences of A. hydrophila AC133 and AC185 were deposited in NCBI GenBank database under accession numbers CP093309 and CP093308, respectively.
